# *N* ^4^,*N* ^4′^-Bis(4-meth­oxy­benzyl­idene)-3,3′-dimethyl­benzidine

**DOI:** 10.1107/S1600536811023749

**Published:** 2011-06-22

**Authors:** Juangang Wang, Peipei Yang, Jin Yang

**Affiliations:** a College of Chemistry and Material Science, Huaibei Normal University, Xiangshan, Huaibei 235000, People’s Republic of China

## Abstract

The mol­ecule of the title compund, C_30_H_28_N_2_O_2_, a Schiff base synthesised *via* a condensation reaction between 4-meth­oxy­benzaldehyde and 3,3′-dimethyl­benzidine, a crystallographic twofold rotation axis passes through the mid-point of the C—C bond of the biphenyl unit. Thus, the asymmetric unit comprises one half-mol­ecule. In the biphenyl unit, the aromatic rings are twisted by 13.49 (7)° with respect to one another. The dihedral angles between the biphenyl and meth­oxy­benzene rings are 49.95 (12) and 50.06 (12)°. In the crystal, weak inter­molecular C—H⋯ O hydrogen bonds contribute to the stabilization of the packing.

## Related literature

For the biological properties of Schiff base ligands, see: Bedia *et al.* (2006 ▶). For related structures, see: Harada *et al.* (2004 ▶); Nesterov (2004 ▶). For reference bond-length values, see: Allen *et al.* (1987 ▶).
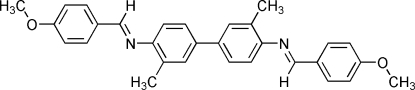


## Experimental

### 

#### Crystal data

C_30_H_28_N_2_O_2_
*M* *_r_* = 448.54
Monoclinic, 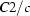

*a* = 11.644 (2) Å
*b* = 16.228 (3) Å
*c* = 12.431 (3) Å
β = 91.75 (3)°
*V* = 2347.9 (8) Å^3^
*Z* = 4
Mo *K*α radiation
μ = 0.08 mm^−1^
*T* = 93 K
0.38 × 0.34 × 0.22 mm

#### Data collection

Rigaku R-AXIS RAPID diffractometer
Absorption correction: multi-scan (*ABSCOR*; Higashi, 1995 ▶) *T*_min_ = 0.703, *T*_max_ = 0.786
11279 measured reflections
2667 independent reflections
2350 reflections with *I* > 2σ(*I*)
*R*_int_ = 0.033

#### Refinement

*R*[*F* ^2^ > 2σ(*F* ^2^)] = 0.039
*wR*(*F* ^2^) = 0.111
*S* = 1.05
2667 reflections
210 parameters
All H-atom parameters refined
Δρ_max_ = 0.40 e Å^−3^
Δρ_min_ = −0.19 e Å^−3^

### 

Data collection: *RAPID-AUTO* (Rigaku, 1998 ▶); cell refinement: *RAPID-AUTO*; data reduction: *CrystalStructure* (Rigaku/MSC, 2004 ▶); program(s) used to solve structure: *SHELXS97* (Sheldrick, 2008 ▶); program(s) used to refine structure: *SHELXL97* (Sheldrick, 2008 ▶); molecular graphics: *XP* in *SHELXTL* (Sheldrick, 2008 ▶); software used to prepare material for publication: *SHELXL97*.

## Supplementary Material

Crystal structure: contains datablock(s) I, global. DOI: 10.1107/S1600536811023749/kp2337sup1.cif

Structure factors: contains datablock(s) I. DOI: 10.1107/S1600536811023749/kp2337Isup2.hkl

Supplementary material file. DOI: 10.1107/S1600536811023749/kp2337Isup3.cml

Additional supplementary materials:  crystallographic information; 3D view; checkCIF report

## Figures and Tables

**Table 1:** Hydrogen-bond geometry (Å, °)

*D*—H⋯*A*	*D*—H	H⋯*A*	*D*⋯*A*	*D*—H⋯*A*
C6—H6*A*⋯O1^i^	0.960 (13)	2.688 (13)	3.4462 (15)	136.32 (su?)

Symmetry code: (i) 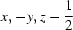.

**Fig. 1.**
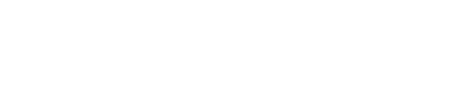
The molecular structure of the title compound, drawn with 30% probability displacement ellipsoids. The molecule has the crystallographic twofold rotation axes through the mid of C12-C12A. To generate the whole molecule symmetry operation A: -x,y, 1/2-z was used.

## supplementary crystallographic information

### Comment

Schiff base ligands have received considerable attention during the last decades, mainly because of their structures or their biological properties (Bedia *et al.*, 2006). We report here the crystal structure of the title compound, (I), a novel Schiff base (Fig. 1). The bond lengths (Allen *et al.*, 1987) and angles are normal and comparable to the values observed in similar compounds (Harada *et al.*, 2004; Nesterov *et al.*, 2004). In the biphenyl moiety, the aromatic rings are inclined by 13.49 (7)°. The dihedral angles between the biphenyl and methoxybenzal rings are 49.95 (12)° and 50.06 (12)°, respectively. In the crystal, weak intermolecular C—H··· O hydrogen bonds contribute to the stabilisation of the packing (Table 1).

### Experimental

4-Methoxybenzaldehyde (0.272 g,2 mmol), 3,3'-Dimethylbenzidine (0.212 g,1 mmol) and 20 mL toluene were placed in a round-bottom flask equipped with a magnetic stirring bar. The reaction mixture was stirred at reflux for 4 h and then cooled.The solid that precipitated was filtered off and washed with methanol. This compound (0.224 g, 0.5 mmol) was dissolved in ethylacetate and left to crystallise. Crystals obtained were suitable for data collection.

### Crystal data

**Table:**

C_30_H_28_N_2_O_2_	*F*(000) = 952
*M**_r_* = 448.54	*D*_x_ = 1.269 Mg m^−^^3^
Monoclinic, *C*2/*c*	Mo *K*α radiation, λ = 0.71073 Å
Hall symbol: -C 2yc	Cell parameters from 9010 reflections
*a* = 11.644 (2) Å	θ = 3.0–27.5°
*b* = 16.228 (3) Å	µ = 0.08 mm^−^^1^
*c* = 12.431 (3) Å	*T* = 93 K
β = 91.75 (3)°	Block, yellow
*V* = 2347.9 (8) Å^3^	0.38 × 0.34 × 0.22 mm
*Z* = 4	

### Data collection

**Table:**

Rigaku R-AXIS RAPID diffractometer	2667 independent reflections
Radiation source: fine-focus sealed tube	2350 reflections with *I* > 2σ(*I*)
graphite	*R*_int_ = 0.033
ω scans	θ_max_ = 27.5°, θ_min_ = 3.0°
Absorption correction: multi-scan (*ABSCOR* ;Higashi, 1995)	*h* = −15→15
*T*_min_ = 0.703, *T*_max_ = 0.786	*k* = −20→20
11279 measured reflections	*l* = −14→16

### Refinement

**Table:**

Refinement on *F*^2^	Primary atom site location: structure-invariant direct methods
Least-squares matrix: full	Secondary atom site location: difference Fourier map
*R*[*F*^2^ > 2σ(*F*^2^)] = 0.039	Hydrogen site location: inferred from neighbouring sites
*wR*(*F*^2^) = 0.111	All H-atom parameters refined
*S* = 1.05	*w* = 1/[σ^2^(*F*_o_^2^) + (0.0658*P*)^2^ + 1.0766*P*] where *P* = (*F*_o_^2^ + 2*F*_c_^2^)/3
2667 reflections	(Δ/σ)_max_ = 0.001
210 parameters	Δρ_max_ = 0.40 e Å^−^^3^
0 restraints	Δρ_min_ = −0.19 e Å^−^^3^

### Special details

**Table:**

Geometry. All e.s.d.'s (except the e.s.d. in the dihedral angle between two l.s. planes) are estimated using the full covariance matrix. The cell e.s.d.'s are taken into account individually in the estimation of e.s.d.'s in distances, angles and torsion angles; correlations between e.s.d.'s in cell parameters are only used when they are defined by crystal symmetry. An approximate (isotropic) treatment of cell e.s.d.'s is used for estimating e.s.d.'s involving l.s. planes.
Refinement. Refinement of *F*^2^ against ALL reflections. The weighted *R*-factor *wR* and goodness of fit *S* are based on *F*^2^, conventional *R*-factors *R* are based on *F*, with *F* set to zero for negative *F*^2^. The threshold expression of *F*^2^ > σ(*F*^2^) is used only for calculating *R*-factors(gt) *etc*. and is not relevant to the choice of reflections for refinement. *R*-factors based on *F*^2^ are statistically about twice as large as those based on *F*, and *R*- factors based on ALL data will be even larger.

### Fractional atomic coordinates and isotropic or equivalent isotropic displacement parameters (Å^2^)

**Table:**

	*x*	*y*	*z*	*U*_iso_*/*U*_eq_	
O1	0.14801 (6)	0.07986 (5)	1.08572 (5)	0.01981 (19)	
N1	0.36979 (7)	0.15477 (5)	0.62842 (7)	0.0176 (2)	
C1	0.20415 (11)	0.12644 (8)	1.16991 (9)	0.0262 (3)	
H1A	0.2839 (14)	0.1056 (10)	1.1839 (12)	0.034 (4)*	
H1C	0.2050 (14)	0.1847 (10)	1.1522 (12)	0.038 (4)*	
H1B	0.1570 (13)	0.1177 (10)	1.2330 (12)	0.037 (4)*	
C2	0.18777 (9)	0.08948 (6)	0.98408 (8)	0.0165 (2)	
C3	0.28693 (9)	0.13363 (6)	0.96025 (8)	0.0183 (2)	
H3A	0.3342 (12)	0.1589 (8)	1.0165 (11)	0.025 (3)*	
C4	0.31999 (9)	0.13940 (6)	0.85410 (8)	0.0179 (2)	
H4A	0.3903 (12)	0.1681 (8)	0.8377 (10)	0.022 (3)*	
C5	0.25488 (8)	0.10280 (6)	0.77062 (8)	0.0163 (2)	
C6	0.15643 (9)	0.05837 (6)	0.79627 (8)	0.0179 (2)	
H6A	0.1119 (11)	0.0334 (8)	0.7388 (10)	0.021 (3)*	
C7	0.12313 (9)	0.05104 (6)	0.90185 (8)	0.0179 (2)	
H7A	0.0561 (12)	0.0194 (8)	0.9196 (10)	0.024 (3)*	
C8	0.28718 (8)	0.10911 (6)	0.65788 (8)	0.0176 (2)	
H8A	0.2396 (11)	0.0762 (8)	0.6043 (10)	0.020 (3)*	
C9	0.40025 (8)	0.15379 (6)	0.51899 (8)	0.0162 (2)	
C10	0.43005 (8)	0.22903 (6)	0.47132 (8)	0.0159 (2)	
C11	0.46879 (8)	0.22851 (6)	0.36602 (8)	0.0165 (2)	
H11A	0.4919 (11)	0.2820 (9)	0.3346 (10)	0.023 (3)*	
C12	0.47841 (8)	0.15556 (6)	0.30593 (7)	0.0160 (2)	
C13	0.44641 (9)	0.08169 (6)	0.35559 (8)	0.0180 (2)	
H13A	0.4501 (11)	0.0292 (8)	0.3175 (10)	0.019 (3)*	
C14	0.40833 (9)	0.08084 (6)	0.46040 (8)	0.0183 (2)	
H14A	0.3897 (12)	0.0279 (8)	0.4927 (10)	0.024 (3)*	
C15	0.41802 (9)	0.30788 (7)	0.53365 (8)	0.0199 (2)	
H15C	0.3378 (12)	0.3186 (9)	0.5538 (11)	0.028 (3)*	
H15B	0.4624 (13)	0.3043 (9)	0.6036 (12)	0.032 (4)*	
H15A	0.4450 (12)	0.3550 (8)	0.4925 (11)	0.025 (3)*	

### Atomic displacement parameters (Å^2^)

**Table:**

	*U*^11^	*U*^22^	*U*^33^	*U*^12^	*U*^13^	*U*^23^
O1	0.0209 (4)	0.0261 (4)	0.0126 (4)	−0.0009 (3)	0.0023 (3)	0.0020 (3)
N1	0.0179 (4)	0.0209 (4)	0.0141 (4)	0.0005 (3)	0.0020 (3)	0.0021 (3)
C1	0.0341 (6)	0.0295 (6)	0.0151 (5)	−0.0047 (5)	0.0020 (4)	−0.0012 (4)
C2	0.0178 (5)	0.0179 (5)	0.0140 (5)	0.0039 (4)	0.0024 (3)	0.0029 (4)
C3	0.0184 (5)	0.0198 (5)	0.0166 (5)	−0.0003 (4)	−0.0011 (4)	0.0006 (4)
C4	0.0163 (5)	0.0192 (5)	0.0181 (5)	−0.0012 (4)	0.0012 (4)	0.0023 (4)
C5	0.0166 (5)	0.0171 (5)	0.0153 (5)	0.0021 (4)	0.0018 (3)	0.0019 (4)
C6	0.0171 (5)	0.0201 (5)	0.0166 (5)	−0.0001 (4)	0.0005 (4)	−0.0009 (4)
C7	0.0160 (5)	0.0195 (5)	0.0185 (5)	−0.0010 (4)	0.0032 (4)	0.0010 (4)
C8	0.0168 (5)	0.0200 (5)	0.0160 (5)	0.0009 (4)	0.0003 (4)	0.0014 (4)
C9	0.0130 (4)	0.0224 (5)	0.0132 (5)	−0.0003 (4)	0.0003 (3)	0.0011 (4)
C10	0.0129 (4)	0.0197 (5)	0.0150 (5)	−0.0008 (3)	0.0000 (3)	−0.0005 (4)
C11	0.0161 (5)	0.0184 (5)	0.0152 (5)	−0.0007 (4)	0.0008 (3)	0.0015 (4)
C12	0.0151 (5)	0.0191 (5)	0.0138 (5)	0.0002 (3)	0.0003 (4)	0.0004 (4)
C13	0.0195 (5)	0.0179 (5)	0.0167 (5)	−0.0003 (4)	0.0009 (4)	−0.0012 (4)
C14	0.0186 (5)	0.0187 (5)	0.0176 (5)	−0.0014 (4)	0.0016 (4)	0.0032 (4)
C15	0.0223 (5)	0.0203 (5)	0.0172 (5)	−0.0017 (4)	0.0040 (4)	−0.0021 (4)

### Geometric parameters (Å, °)

**Table:**

O1—C2	1.3677 (12)	C7—H7A	0.965 (14)
O1—C1	1.4325 (14)	C8—H8A	1.006 (13)
N1—C8	1.2767 (13)	C9—C14	1.3946 (15)
N1—C9	1.4164 (12)	C9—C10	1.4054 (14)
C1—H1A	0.999 (16)	C10—C11	1.3976 (14)
C1—H1C	0.972 (17)	C10—C15	1.5046 (14)
C1—H1B	0.981 (15)	C11—C12	1.4061 (14)
C2—C7	1.3978 (15)	C11—H11A	0.992 (14)
C2—C3	1.3982 (14)	C12—C13	1.4039 (14)
C3—C4	1.3891 (14)	C12—C12^i^	1.4930 (18)
C3—H3A	0.968 (14)	C13—C14	1.3892 (14)
C4—C5	1.3987 (15)	C13—H13A	0.975 (13)
C4—H4A	0.969 (14)	C14—H14A	0.976 (13)
C5—C6	1.3993 (14)	C15—H15C	0.990 (14)
C5—C8	1.4657 (13)	C15—H15B	1.000 (15)
C6—C7	1.3851 (14)	C15—H15A	0.977 (14)
C6—H6A	0.960 (13)		
			
C2—O1—C1	117.07 (8)	N1—C8—H8A	121.5 (7)
C8—N1—C9	118.82 (9)	C5—C8—H8A	116.3 (7)
O1—C1—H1A	110.5 (9)	C14—C9—C10	119.73 (9)
O1—C1—H1C	110.8 (9)	C14—C9—N1	122.27 (9)
H1A—C1—H1C	110.7 (13)	C10—C9—N1	117.87 (9)
O1—C1—H1B	104.7 (9)	C11—C10—C9	118.72 (9)
H1A—C1—H1B	110.6 (12)	C11—C10—C15	121.69 (9)
H1C—C1—H1B	109.4 (13)	C9—C10—C15	119.58 (9)
O1—C2—C7	115.77 (9)	C10—C11—C12	122.41 (9)
O1—C2—C3	124.00 (9)	C10—C11—H11A	117.6 (7)
C7—C2—C3	120.23 (9)	C12—C11—H11A	120.0 (7)
C4—C3—C2	119.37 (10)	C13—C12—C11	117.30 (9)
C4—C3—H3A	119.3 (8)	C13—C12—C12^i^	120.73 (6)
C2—C3—H3A	121.3 (8)	C11—C12—C12^i^	121.96 (6)
C3—C4—C5	121.10 (9)	C14—C13—C12	121.17 (9)
C3—C4—H4A	119.5 (8)	C14—C13—H13A	117.8 (7)
C5—C4—H4A	119.4 (8)	C12—C13—H13A	121.1 (7)
C4—C5—C6	118.62 (9)	C13—C14—C9	120.66 (9)
C4—C5—C8	122.06 (9)	C13—C14—H14A	118.4 (7)
C6—C5—C8	119.33 (9)	C9—C14—H14A	120.9 (7)
C7—C6—C5	121.02 (10)	C10—C15—H15C	112.6 (8)
C7—C6—H6A	120.6 (8)	C10—C15—H15B	110.1 (9)
C5—C6—H6A	118.4 (8)	H15C—C15—H15B	104.9 (11)
C6—C7—C2	119.64 (9)	C10—C15—H15A	111.0 (8)
C6—C7—H7A	120.9 (8)	H15C—C15—H15A	108.4 (11)
C2—C7—H7A	119.4 (8)	H15B—C15—H15A	109.6 (12)
N1—C8—C5	122.17 (9)		

Symmetry codes: (i) −*x*+1, *y*, −*z*+1/2.

### Hydrogen-bond geometry (Å, °)

**Table:**

*D*—H···*A*	*D*—H	H···*A*	*D*···*A*	*D*—H···*A*
C6—H6A···O1^ii^	0.960 (13)	2.688 (13)	3.4462 (15)	136.32

Symmetry codes: (ii) *x*, −*y*, *z*−1/2.
